# AdipoRon’s Impact on Alzheimer’s Disease—A Systematic Review and Meta-Analysis

**DOI:** 10.3390/ijms26020484

**Published:** 2025-01-08

**Authors:** Sandra Maria Barbalho, Lucas Fornari Laurindo, Bárbara de Oliveira Zanuso, Rebeca Maria Siqueira da Silva, Lívia Gallerani Caglioni, Victor Bruno Fonseca Nunes Junqueira de Moraes, Lívia Fornari Laurindo, Victória Dogani Rodrigues, Jéssica da Silva Camarinha Oliveira, Maria Eduarda Beluce, Cláudia Rucco Penteado Detregiachi, Caroline Barbalho Lamas, Jesselina Francisco dos Santos Haber, Virgínia Maria Cavallari Strozze Catharin, Karina Quesada, Masaru Tanaka, Vitor Engrácia Valenti

**Affiliations:** 1 Department of Biochemistry and Pharmacology, School of Medicine, Universidade de Marília (UNIMAR), Marília 17525-902, São Paulo, Brazil; smbarbalho@gmail.com (S.M.B.); lucasffffor@gmail.com (L.F.L.);; 2 Postgraduate Program in Structural and Functional Interactions in Rehabilitation, School of Medicine, Universidade de Marília (UNIMAR), Marília 17525-902, São Paulo, Brazil; 3 Department of Biochemistry and Nutrition, School of Food and Technology of Marília (FATEC), Marília 17500-000, São Paulo, Brazil; 4 UNIMAR Charity Hospital, Universidade de Marília (UNIMAR), Marília 17525-902, São Paulo, Brazil; 5 Department of Biochemistry and Pharmacology, School of Medicine, Faculdade de Medicina de Marília (FAMEMA), Marília 17519-030, São Paulo, Brazil; 6 Department of Administration, Associate Degree in Hospital Management, Universidade de Marília (UNIMAR), Marília 17525-902, São Paulo, Brazil; 7 Department of Biochemistry and Pharmacology, School of Medicine, Faculdade de Medicina de São José do Rio Preto (FAMERP), São José do Rio Preto 15090-000, São Paulo, Brazil; 8 Department of Gerontology, School of Gerontology, Universidade Federal de São Carlos (UFSCar), São Carlos 13565-905, São Paulo, Brazil; 9 Danube Neuroscience Research Laboratory, HUN-REN-SZTE Neuroscience Research Group, Hungarian Research Network, University of Szeged (HUN-REN-SZTE), Tisza Lajos Krt. 113, H-6725 Szeged, Hungary; 10 Autonomic Nervous System Center, School of Philosophy and Sciences, São Paulo State University, Marília 17525-902, São Paulo, Brazil

**Keywords:** AdipoRon, Alzheimer’s disease, adiponectin, cognitive impairment, synaptic defects, neurology, adiponectin replacement therapy, adiponectin receptor agonist

## Abstract

Alzheimer’s disease (AD) remains a leading cause of cognitive decline and mortality worldwide, characterized by neurodegeneration, synaptic deficiencies, and neuroinflammation. Despite advancements in early detection, diagnosis, and treatment, AD presents substantial challenges due to its complex pathology, heterogeneity, and the limited efficacy of current therapies. Consequently, there is a pressing need for novel therapeutic agents to target the multifaceted aspects of AD pathology, enhance current treatments, and minimize adverse effects. AdipoRon, an adiponectin receptor agonist, has garnered interest for its potential neuroprotective effects, including reducing neuroinflammation, improving mitochondrial function, and mitigating tau hyperphosphorylation. This review aimed to evaluate the effects of AdipoRon-based adiponectin replacement therapy against AD, using a comprehensive approach grounded in the PICO framework—Population, Intervention, Comparison, and Outcomes. A total of six studies were reviewed, including in vitro and in vivo investigations examining AdipoRon’s impact on various AD models. These studies involved different cell lines and transgenic mouse models, assessing various outcomes such as cognitive function, neuroinflammation, tau phosphorylation, synaptic deficiencies, and relevant molecular pathways. By synthesizing data from these studies, our review thoroughly explains AdipoRon’s neuroprotective effects, mechanisms of action, and potential as a therapeutic agent for AD. This analysis aims to highlight the current state of knowledge, identify gaps in the research, and suggest directions for future studies and clinical applications.

## 1. Introduction

Adipokines, secreted by the adipose tissue, are bioactive molecules that play an essential role in inflammation and metabolism regulation, energy homeostasis, cardiovascular function, immunity, and diseases such as cancer and dementia. However, as two-sided coins, adipokines may contribute to obesity-related disorders during adipose tissue dysfunction. The paramount understanding of adipokines relies on the scenario of their release. In healthy adipose tissue individuals, adipokines are mainly anti-inflammatory. In dysfunctional adipose tissue subjects, adipokines are mostly pro-inflammatory. While anti-inflammatory adipokines decrease, the likelihood of cardiovascular and other inflammatory metabolic diseases increases [1,2,3,4]. Obesity induces an imbalance of adipokines. In obese individuals, “good” adipokines such as adiponectin and omentin-1 are down-regulated. These adipokines maintain the survival and functional plasticity of mesenchymal stem cells. Conversely, “bad” adipokines such as leptin, visfatin, and resistin also promote mesenchymal stem cells to differentiate into adipocytes, impairing pleiotropic functions and accelerating senescence and cell death via apoptosis [5,6,7]. In the brain, receptors for adipokines are abundantly presented. Evidence has demonstrated that adipokines such as adiponectin cross the blood-brain barrier, and it is known that the neurotrophic and anti-inflammatory properties of adipokines drive their neuroprotective effects against neurodegeneration [8,9,10,11,12,13]. Investigations reported that dysregulated adipokine secretion underlines the co-occurrence of comorbidities such as metabolic syndromes and neurodegenerative diseases [14,15,16,17,18,19,20,21,22].

Alzheimer’s disease (AD) stands as one of the most pressing challenges in neurodegenerative disorders, characterized by a relentless decline in cognitive function and the accumulation of pathological proteins such as Amyloid-Beta (Aβ) plaques and hyperphosphorylated tau tangles [23,24,25,26,27,28]. This complex and progressive disease disrupts daily functioning and quality of life for millions worldwide, underscoring the urgent need for innovative and effective treatment strategies [29,30,31].

In recent years, AD research has increasingly focused on novel therapeutic approaches targeting underlying metabolic and neurodegenerative processes [32,33,34,35,36]. One promising strategy involves AdipoRon, a synthetic agonist that activates the adiponectin receptor [37,38,39,40]. Adiponectin is a hormone secreted by adipose tissue that is well-regarded for its role in regulating metabolic processes such as glucose homeostasis and fatty acid oxidation [41,42,43,44]. Beyond its metabolic functions, adiponectin has garnered attention for its potential neuroprotective effects, which have been linked to its ability to influence several critical pathways involved in neurodegeneration [45,46,47,48].

Adiponectin, AdipoQ, or ACRP30 has a 244-amino acid structure and a molecular weight of approximately 26 kDa. Regarding abundance, adiponectin is the second most secreted hormone in the brain. Therefore, many studies have indicated that alterations in adiponectin secretion are related to adipose tissue dysregulation and obesity-related diseases. Regarding mechanisms of action, adiponectin influences metabolism, playing a crucial role in modulating energy homeostasis, inflammation, and other intricate cellular processes such as insulin sensitivity. Adiponectin possesses an N-terminal sequence, a C-terminal domain, a hypervariable domain, and 15 collagenous repeats. The high molecular weight is the most biologically active isoform, which is also the most encountered in plasma [31,49,50]. Adiponectin exerts its effects through various receptors, including Adiponectin Receptor 1 (AdipoR1) and Adiponectin Receptor 2 (AdipoR2), which are involved in modulating critical cellular processes [50,51]. These receptors activate downstream signaling pathways that regulate inflammation, oxidative stress, and mitochondrial function—crucial factors in neurodegenerative diseases like Alzheimer’s [52,53]. Adiponectin’s actions include enhancing insulin sensitivity [54,55,56,57], reducing inflammation [55,56,58,59,60,61], and promoting neuronal survival [62,63,64], all of which are beneficial for maintaining brain health and function [65,66,67,68,69]. Beyond AdipoR1 and AdipoR2, adiponectin also binds to T-cadherin. This third adiponectin receptor is a glycosyl phosphatidylinositol-anchored protein. T-cadherin is most highly expressed within the cardiovascular system, composed of a pro-peptide and five distinct extracellular cadherin domains. This protein lacks transmembrane and cytoplasmic regions. Besides its complex structure, T-cadherin only binds to the hexametric and high molecular weight adiponectin isoforms, but not trimeric or spherical forms [70]. However, studies elucidated that adiponectin binding to T-cadherin protects against neointima proliferation and atherosclerosis, which protects individuals against dementia since atherosclerosis is intimately associated with the occurrence of AD [71,72].

**Figure 1:**
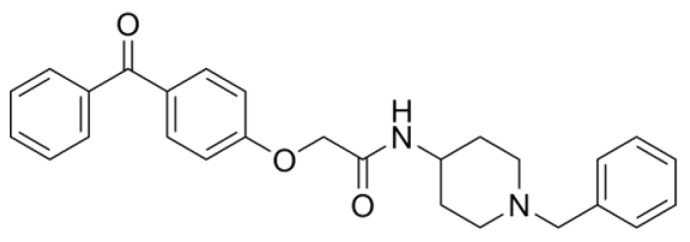
Molecular Structure of AdipoRon. AdipoRon, with the molecular formula C_27_H_28_N_2_O_3_, has a molecular weight of 428.5 g/mol. Its exact mass is 428.20999276 Da, aligning with its monoisotopic mass. The compound exhibits high lipophilicity, with an XLogP3-AA value of 4.7, indicating it is more soluble in fats than water. It has one hydrogen bond donor and four hydrogen bond acceptors, suggesting its capacity for hydrogen bonding and potential interactions with other molecules. AdipoRon features eight rotatable bonds, reflecting its structural flexibility. Its topological polar surface area is 58.6 Å^2^, suggesting moderate polarity, influencing its ability to cross biological membranes. With a heavy atom count of 32, AdipoRon is relatively complex, and its complexity value of 582 further supports this. The compound is electrically neutral with a formal charge of 0 and lacks chiral centers, indicating no stereoisomerism. These properties highlight AdipoRon’s lipophilic nature, structural complexity, and potential behavior in biological contexts [91].

AdipoRon (Figure 1), a synthetic adiponectin receptor agonist, was developed to replicate and amplify these beneficial effects [73,74,75,76,77,78]. AdipoRon is believed to harness its therapeutic potential for treating AD by mimicking the adiponectin action. Preclinical studies have shown that AdipoRon can activate similar signaling pathways to adiponectin, such as the Adenosine Monophosphate-Activated Protein Kinase (AMPK) pathway [74,77,79,80,81,82], which plays a role in regulating energy metabolism and cellular stress responses [83,84]. Additionally, AdipoRon has been observed to influence autophagy [31,82,85,86,87,88], a process critical for clearing neurotoxic proteins and maintaining cellular homeostasis [27,89,90].

This approach is auspicious due to the multifaceted role of adiponectin in neuroprotection. AdipoRon, by modulating critical pathways involved in cellular stress responses and metabolism, presents a potential therapeutic strategy for addressing the complex pathology of AD [92,93]. By intervening in these fundamental mechanisms, AdipoRon holds the potential to slow disease progression, improve cognitive function, and support overall brain health. This makes AdipoRon a compelling option for advancing AD treatment and enhancing patient outcomes. Given these insights, this article presents a comprehensive systematic review of AdipoRon-based therapies for AD. To our knowledge, this is the only review that has thoroughly explored the full potential of AdipoRon in this context. We examine a broad array of studies using both in vitro and in vivo models to uncover the therapeutic benefits and mechanisms of AdipoRon treatment. Our review will detail experimental designs, treatment regimens, and outcomes from various studies, highlighting how AdipoRon addresses both the cognitive and pathological features of AD. Additionally, we will discuss the possible clinical implications of each finding and outline future research endeavors needed to investigate further and validate AdipoRon’s therapeutic potential.

## 2. Materials and Methods

### 2.1. Database Search

A comprehensive literature review evaluated the effects of AdipoRon-based adiponectin replacement therapy against AD. Multiple databases, including PubMed, Scopus, Web of Science, and Google Scholar, were systematically searched using the PICO framework—Population, Intervention, Comparison, and Outcomes. Keywords included “AdipoRon”, “adiponectin replacement therapy”, “Alzheimer’s disease”, “in vitro”, “in vivo”, “neuroinflammation”, “tau hyperphosphorylation”, “cognitive function”, “mitochondrial fusion”, and “autophagy”. Boolean operators like AND, OR, and NOT were utilized to refine the search results, for example, “AdipoRon AND Alzheimer’s disease” or “AdipoRon AND neurodegeneration”. Filters such as publication date (last decade), language (English), and document type (original research articles) were applied to ensure the relevance and quality of the studies included. The search strategy aimed to capture a broad range of studies focusing on AdipoRon-based therapies in AD treatment, providing a comprehensive review of the available evidence.

### 2.2. Inclusion Criteria

Due to the absence of clinical trials, the inclusion criteria for the studies were explicitly focused on preclinical research. These included studies involving either in vitro or in vivo models of AD that featured experimental groups treated with AdipoRon. We required that the studies reported outcomes related to cognitive function, neuroinflammation, tau phosphorylation, synaptic deficiencies, and relevant molecular pathways to ensure a comprehensive evaluation of AdipoRon’s effects against AD since these are key features for AD development. Studies must have clear and robust experimental designs with a detailed methodology, result reporting, and enough description between article elements (tables, figures, and images) and the manuscript’s main text. They had to be published in peer-reviewed journals and provide detailed information on the intervention, control, endpoints, results, and conclusions. Only articles published in the last decade were considered to ensure the relevance of the findings to current research and advancements. Additionally, only studies published in English were included to maintain consistency and ensure accurate interpretation of the data. Discrepancies between the authors on what articles to include were to be resolved by the two authors with the most publications on AdipoRon through a critical and conjunct assessment of the study based on the inclusion criteria. Fortunately, there were no discrepancies during the selection process. These two authors have direct communication.

### 2.3. Exclusion Criteria

Exclusion criteria included reviews, meta-analyses, non-experimental papers, studies not involving AdipoRon as an intervention, and those not based on AD models. Studies with inadequate experimental designs or unclear reporting of results were also excluded.

### 2.4. Data Extraction

Data extraction was performed using a standardized form to capture essential information on experimental models, AdipoRon treatment details (concentration and duration), outcomes, and limitations. Results were detailed within a table, and all the table elements were designed according to the PICO framework (study type and models, experimental grouping and treatment options/durations/interventions, outcomes, and relevant pathways) to ensure rigor and conciseness, meeting systematic reviews’ standards.

### 2.5. Quality Assessment

The quality of the studies was assessed based on their experimental design, sample size, and clarity of result reporting, following the established PRISMA guidelines for scientific rigor. Initially, the methodological rigor of each study was evaluated based on the design and execution of experiments, the appropriateness of models and controls, and the clarity in defining interventions and endpoints. The sample size was examined to ensure it was adequate for drawing reliable conclusions, and the comprehensiveness of data reporting was assessed to verify that the results were presented with sufficient detail. Potential biases were identified, including variability in experimental conditions and short-term versus long-term outcomes. The studies’ ability to translate preclinical findings into potential clinical implications received particular attention. A qualitative data synthesis was conducted to summarize the effects of AdipoRon on AD models, identify expected outcomes, and discuss limitations. The findings were organized to highlight the therapeutic effects of AdipoRon, its mechanisms of action, and its potential as a treatment for AD. By systematically applying these criteria, we aimed to ensure a thorough and objective evaluation of the studies’ overall quality and relevance to advancing AdipoRon-based AD therapies. This review also aimed to identify gaps in current research and suggest directions for future studies and clinical applications.

### 2.6. Synthesis of Results and Summary Measures

After selecting all references, we evaluated the possibility of meta-analysis. The information required to construct the meta-analysis was from the post-intervention period. We adopted the criterion of extracting all data offered between groups post-intervention. Heterogeneity was calculated via the I^2^ statistics. We interpreted 0–40% as unimportant, 30–60% as moderate heterogeneity, 50–75% as substantial heterogeneity, and 75–100% as considerable heterogeneity [94,95]. For the “95% CI” and “Test for overall effect size” values, significant differences were assumed for *p* < 0.05 (or <5%). If the studies did not provide dispersion values of change such as Standard Deviation (SD), 95% CI, standard errors, or *p*-values, the missing SD of the changes (SDchanges) were calculated. Outcomes of the meta-analysis were reported in weighted MD, 95% CI, and *p*-values. *p* < 0.05 were considered statistically significant for the overall MD of the intervention group compared to the control group. The results were presented in forest plots. We imposed a random-effect model, as this more conservative method permitted the study heterogeneity to deviate beyond chance, providing further generalizable results [96]. All data were formed using the Review Manager Program (RevMan 5.4.1).

## 3. Results

**Figure 2:**
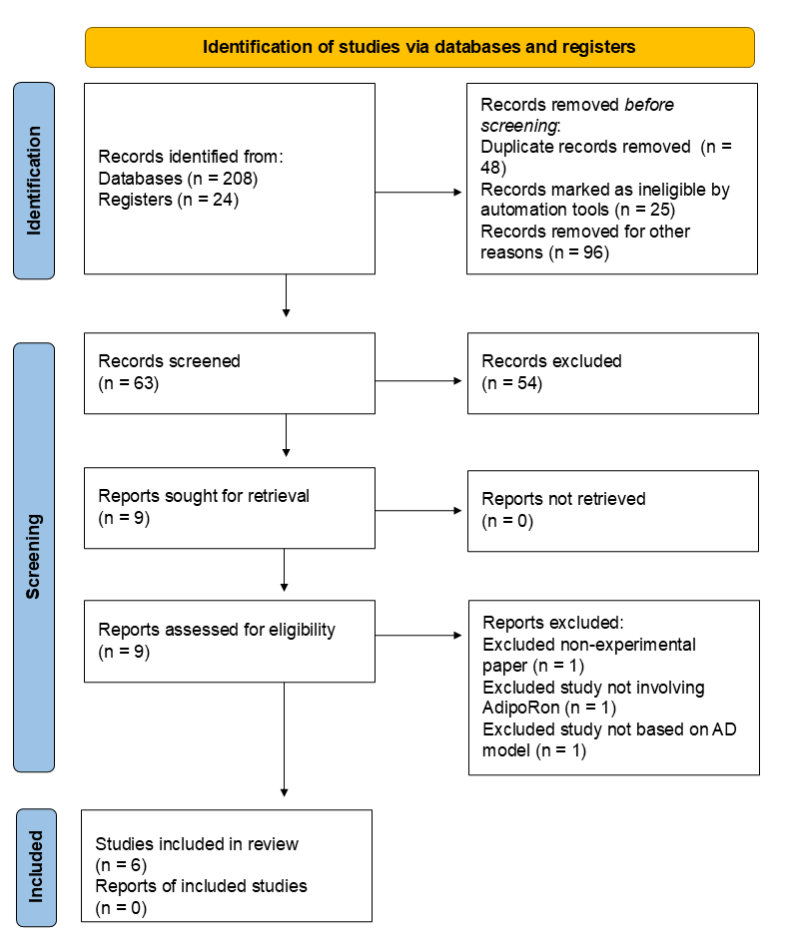
Preferred Reporting Items for Systematic Reviews and Meta-Analyses (PRISMA) flow diagram depicting the systematic review process. AD, Alzheimer’s disease.

Figure 2 illustrates the Preferred Reporting Items for Systematic Reviews and Meta-Analyses (PRISMA) [97] flow diagram for the review process. The database search was conducted between 1 July 2024 and 1 November 2024. The search process yielded 208 records from databases and 24 records from registers. Following this initial identification, duplicate records were systematically removed, totaling 48. Additionally, 25 records were flagged as ineligible by automated tools (Rayyan automated tool, https://www.rayyan.ai/, accessed on 31 October 2024), and a further 96 records were excluded for various reasons deemed appropriate (54 due to publication type, 24 for language restrictions, and 18 for inappropriate study design). After these exclusions, 63 records remained for manual screening. This process resulted in the exclusion of 54 records that needed to meet the criteria for further review. Subsequently, nine reports were requested for retrieval to gather more detailed information. Fortunately, all nine reports were successfully retrieved. Each of the nine successfully obtained reports was carefully assessed for eligibility. This assessment led to the exclusion of three reports for the following reasons: one was a non-experimental paper, one did not involve AdipoRon, and one was not based on an AD model. In the end, six studies were deemed eligible and included in the review. However, no additional reports related to the studies included were available.

Table 1 outlines the effects of AdipoRon-based adiponectin replacement therapy on AD, including the study types, experimental models, treatment regimens, outcomes, and involved pathways. It highlights its impact on cognitive function, Aβ deposition, neuroinflammation, and the molecular mechanisms through which AdipoRon exerts its effects.

**Figure 3:**
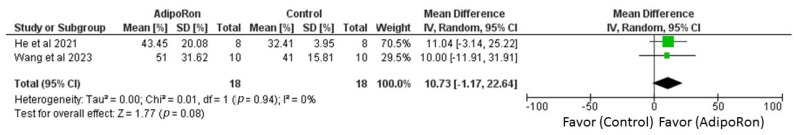
Meta-analysis for overall effects of AdipoRon on novel object preference [99,102].

**Figure 4:**
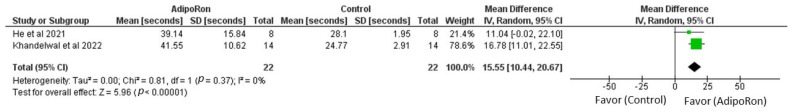
Meta-analysis for overall effects of AdipoRon on time spent in the novel arm [100,102].

**Figure 5:**
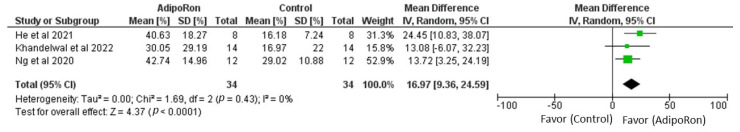
Meta-analysis for overall effects of AdipoRon on time spent in the target quadrant [100,101,102].

**Figure 6:**
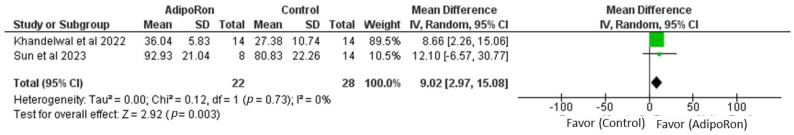
Meta-analysis for overall effects of AdipoRon on total arm entries [98,100].

**Figure 7:**
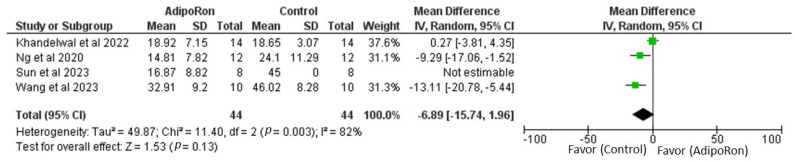
Meta-analysis for overall effects of AdipoRon on escape latency [98,99,100,101].

**Figure 8:**
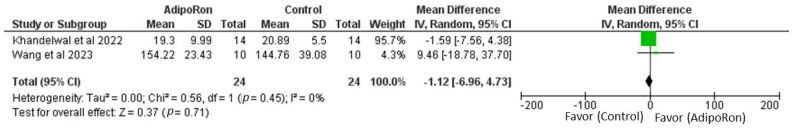
Meta-analysis for overall effects of AdipoRon on swimming speed [99,100].

**Figure 9:**
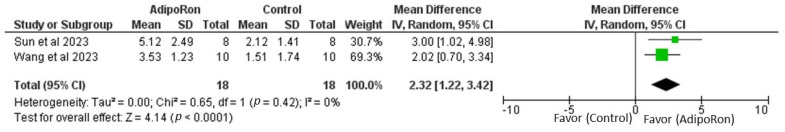
Meta-analysis for overall effects of AdipoRon on crossing times [98,99].

Figure 3 summarizes data from studies evaluating the effect of AdipoRon versus control on novel object preference. The pooled Mean Difference (MD) favors AdipoRon, though the overall effect does not reach statistical significance (MD = 10.73, 95% CI [−1.17, 22.64]; *p* = 0.08). The heterogeneity is negligible (I^2^ = 0%). Figure 4 illustrates the results of studies comparing AdipoRon-treated groups and controls for the time spent in the novel arm during behavioral testing. The pooled MD significantly favors AdipoRon (MD = 15.55, 95% CI [10.44, 20.67]; *p* < 0.00001), with no observed heterogeneity (I^2^ = 0%). Figure 5 analyses the effects of AdipoRon on the time spent in the target quadrant in behavioral tasks. The pooled MD significantly favors AdipoRon (MD = 16.97, 95% CI [9.36, 24.59]; *p* < 0.0001), with minimal heterogeneity (I^2^ = 0%). Figure 6, which investigated total arm entries between AdipoRon and control groups, shows a significant overall effect favoring AdipoRon (MD = 9.02, 95% CI [2.97, 15.08]; *p* = 0.003). Heterogeneity is low (I^2^ = 0%). Figure 7 shows escape latency results across studies. The pooled MD does not significantly favor either group (MD = −6.89, 95% CI [−15.74, 1.96]; *p* = 0.13), and high heterogeneity is observed (I^2^ = 82%). Figure 8 examines the swimming speed in behavioral testing. The overall MD suggests no significant difference between AdipoRon and control groups (MD = −1.12, 95% CI [−6.96, 4.73]; *p* = 0.71), with no heterogeneity (I^2^ = 0%). Figure 9 analyses the number of crossing times. AdipoRon shows a significant effect compared to the control (MD = 2.32, 95% CI [1.22, 3.42]; *p* < 0.0001), with no heterogeneity (I^2^ = 0%).

## 4. Discussion

To understand AdipoRon’s potential in addressing AD, it is essential to systematically evaluate its effects through a comprehensive review of existing research. The following analysis aims to uncover the underlying logic behind AdipoRon’s impact and provide a clear assessment of its efficacy and mechanisms of action.

### 4.1. Physicochemical Properties, Structural Characteristics, and Therapeutic Potential of AdipoRon: Progress and Obstacles

Since its discovery in 2013, the initially speculative therapeutic potential of AdipoRon has been confirmed through substantial research. After a thorough screening process at the Open Innovation Center for Drug Discovery (The University of Tokyo), AdipoRon has been identified as a pioneering orally active adiponectin receptor agonist. This recognition stems from its exceptional ability to activate AMPK and bind to AdipoR1 and AdipoR2, as demonstrated in C2C12 murine myoblast cells [103]. Enamine Ltd. (Kiev, Ukraine) developed AdipoRon via a multi-step synthesis, starting with the alkylation of hydroxybenzophenone using methyl chloroacetate. The resulting compound features three distinct functional groups: a 1-benzyl 4-substituted 6-membered cyclic amine moiety, a carbonyl group, and a terminal aromatic ring. Radioactive binding and Scatchard analysis have confirmed the specificity of AdipoRon in vitro, showing its affinity for AdipoR1 and AdipoR2 with dissociation constants (Kd) of approximately 1.8 μM and 3.1 μM, respectively; however, no randomized clinical trials involve AdipoRon in human subjects [73].

Although AdipoRon has been found to promote neuroprotection and the restoration of synaptic functions in various models of brain disease in mice, caution is needed to overcome the limitations associated with AdipoRon use during preclinical practice. Previous research noted that while low-dose AdipoRon treatment enhanced hippocampal cell proliferation while increasing serum adiponectin and the Brain-Derived Neurotrophic Factor (BDNF) in mice with hippocampal defects, higher doses of AdipoRon treatments conversely suppressed hippocampal neurogenesis and influenced reductions in serum levels of adiponectin and BDNF. Thus, it is worth noting that negative feedback may occur by reducing adiponectin secretion while suppressing adipogenesis to overcome the hyperactivation of adiponectin receptors and their mediated cascades, impairing hippocampal neurogenesis. The authors potentially link AdipoRon hyperactivation of AMPK signaling with Mammalian Target of Rapamycin (mTOR) inhibition and BDNF level reduction, consequently leading to impaired hippocampal neurogenesis [104]. Given this scenario and AdipoRon being a lipophilic drug, dose- and time-dependent treatments must be accurately described before AdipoRon can be included in translational medicine to treat neurodegenerative diseases. The Blood-Brain Barrier (BBB) is a protective membrane naturally occurring in the central nervous system. Protecting the brain from toxins and pathogens in the blood, the BBB is crucial for developing the central nervous system and its well-functioning. However, the BBB also possesses complications since it complicates pharmacotherapy for brain disorders [105]. AdipoRon, as a lipophilic drug candidate, has facilitated entering the central nervous system via the BBB. Lipophilic drugs often pass the BBB compartment by passive diffusion or become solubilized into the lipid bilayer of the BBB blood vessels [106]. Although preoccupations such as insufficient drug delivery into the brain may not necessarily be a problem regarding AdipoRon administration in diseases like dementia due to its possible facilitated transport into the central nervous system, we must highlight that more pharmacodynamics and pharmacokinetics studies are necessary to fully realize the potential of AdipoRon in treating brain disorders. Since previous research denoted the toxicity of this synthetic peptide in the realm of hippocampal neurogenesis, caution is warranted in choosing the correct doses and treatment duration regimens.

### 4.2. Evaluating the Therapeutic Potential of AdipoRon in Alzheimer’s Disease

This study evaluates the efficacy of AdipoRon, an adiponectin receptor agonist, in addressing AD, cognitive impairment, and synaptic defects through various in vitro and in vivo models. The results indicate the promising neuroprotective effects of AdipoRon, mediated through several molecular pathways. However, each study has its limitations, which must be considered when interpreting these findings.

Sun et al. [98] explored how AdipoRon affects autophagy in HT22 cells and APP/PS1 transgenic mice, revealing its potential to enhance Aβ clearance and improve cognitive function in AD. The study found that AdipoRon promotes Aβ clearance by activating neuronal autophagy through the AdipoR1/AMPK-dependent nuclear translocation of Glyceraldehyde-3-Phosphate Dehydrogenase (GAPDH) and subsequent activation of Sirtuin1 (SIRT1). This interaction leads to the release of Deleted in Breast Cancer 1 (DBC1) and the activation of SIRT1, which induces autophagy. The inhibition of either GAPDH or SIRT1 counteracts these effects, underscoring the critical role of SIRT1 in AdipoRon’s mechanism of action. Clinically, these findings suggest that AdipoRon could be developed as a therapeutic agent targeting autophagic pathways to enhance Aβ clearance in Alzheimer’s patients, potentially modifying disease progression by targeting underlying pathophysiological mechanisms. This highlights its potential to alleviate symptoms and address the root cause of Aβ accumulation. However, the study’s limitations lie in the reliance on specific mouse models, which may not fully represent the complexity of human AD, necessitating further validation in more diverse models and eventual clinical trials.

Wang et al. [99] conducted experiments using human neuroblastoma cells (SH-SY5Y) and P301S transgenic male mice. AdipoRon treatment reduced hyperphosphorylated tau accumulation and improved memory, synaptic function, and mitochondrial fusion. These effects were mediated through activating the AMPK/Glycogen Synthase Kinase 3 Beta (GSK3β) pathway and increased mitochondrial fusion proteins. Clinically, these findings suggest that AdipoRon could be developed as a therapeutic agent targeting tauopathies and mitochondrial dysfunction in AD. This highlights its potential to alleviate symptoms and modify disease progression by targeting underlying pathophysiological mechanisms. However, a limitation of this study lies in the long-term administration (4 months) in mice, which may not be feasible or directly translatable to human treatment regimens, raising concerns about adherence and long-term safety in a clinical setting. It is crucial to note that long-term AdipoRon administration requires caution. Previous studies have indicated that long-term AdipoRon treatment (20 days) has negatively impacted whole-body insulin sensitivity, increasing Insulin Resistance (IR) through exacerbated adipose tissue lipolysis, increased hepatic gluconeogenesis, and an impaired muscle tricarboxylic acid cycle [79,82,107,108,109,110]. For instance, increased IR increases the likelihood of AD occurrence [111,112,113,114,115,116]. Therefore, translating long-term AdipoRon treatments to humans warrants further attention. This implicates allometric scale studies and safety margins since AdipoRon is the first commercialized adiponectin receptor agonist for preclinical studies in significant proportions. Therefore, comparative approaches with similar drugs are not available. Additionally, human equivalent dose calculators for AdipoRon translation are also not accessible. Translating long-term AdipoRon regimens to humans would certainly raise concerns about metabolic imbalances, hormonal disruptions, or unforeseen toxicities.

Khandelwal et al. [100] studied the effects of AdipoRon on Neuro2A cells and APP/PS1 transgenic male mice. The treatment enhanced glucose uptake and insulin sensitivity in vitro, reducing cognitive deficits, Aβ burden, and neuroinflammation in vivo. These outcomes were achieved by activating Glucose Transporter Protein Type-4 (GLUT4) translocation, Protein Kinase B (AKT), GSK3β, and AMPK phosphorylation pathways. Clinically, this suggests that AdipoRon could mitigate IR and amyloid pathology in Alzheimer’s patients, providing a dual therapeutic benefit by addressing both metabolic dysfunction and amyloid burden. However, using a specific mouse model may limit the applicability of these results to the broader AD population, as different genetic backgrounds and environmental factors in humans could influence treatment efficacy.

Ng et al. [101] explored the impact of AdipoRon on insulin-resistant HT-22 hippocampal cells and 5xFAD mice. The treatment improved neuronal insulin signaling and sensitivity in vitro. AdipoRon also enhanced spatial memory and synaptic function and reduced neuroinflammation in vivo. These benefits were linked to AMPK activation and decreased Beta-Secretase 1 (BACE1) and Nuclear Factor Kappa B (NF-kB) levels. Clinically, AdipoRon holds potential as a therapy for IR-associated cognitive impairment in AD, suggesting a new avenue for treating AD patients with comorbid diabetes or prediabetic conditions. However, another of the study’s limitations is the relatively short treatment period (3 months), which may not capture the long-term effects and safety of the intervention, especially considering the chronic nature of AD.

He et al. [102] used N2a/APPswe cells and multiple mouse models (5xFAD, APP/PS1, and APN KO mice) to assess AdipoRon’s effects. The treatment reduced Aβ deposition, neuroinflammation, and cognitive impairment and enhanced microglia’s autophagic activation and lysosomal activity. These effects were mediated through the AMPK-mTOR pathway and the increased expression of AdipoR1 and Adaptor Protein Phosphotyrosine Interacting with PH Domain and Leucine Zipper 1 (APPL1). Clinically, AdipoRon could be valuable in reducing amyloid pathology and enhancing autophagy in AD patients, potentially slowing disease progression by promoting the clearance of toxic protein aggregates. The limitation here is the diverse genetic backgrounds of the mouse models used, which may lead to a variability in responses that is not representative of the human condition, thereby necessitating further validation in more standardized models and eventual human trials.

Liu et al. [93] investigated the effects of AdipoRon on NE-4C cells and adult male APP/PS1 mice. The treatment improved cell viability and cognitive function and reduced Aβ deposition, mediated by AMPK/Cyclic Adenosine Monophosphate (cAMP)-Response Element Binding Protein (CREB) activation. Clinically, this indicates that AdipoRon could support neuronal survival and cognitive function in Alzheimer’s patients by enhancing neuroplasticity and reducing amyloid toxicity. However, another limitation of this study is the short treatment duration (7 days) in vivo, which may not be sufficient to observe the full spectrum of therapeutic effects. Additionally, the mode of administration (injection) may not be the most practical for long-term treatment in humans, necessitating the development of alternative delivery methods.

The findings from the meta-analysis provide compelling insights into the effects of AdipoRon treatment. Across various behavioral tests, including novel object preference and time spent in the novel arm or target quadrant, AdipoRon significantly improved performance metrics. These outcomes suggest that AdipoRon positively influences spatial memory and cognitive function, supported by pooled mean differences. Results such as increased total arm entries and reduced escape latency in behavioral tasks highlight enhanced task-related motivation and mobility in the AdipoRon-treated groups. This indicates improved physical or neuromuscular performance. Most comparisons show negligible heterogeneity, reinforcing the robustness of the findings. However, high heterogeneity in specific metrics, like escape latency, warrants further investigation into methodological variations or population-specific responses.

Overall, the evidence indicates that AdipoRon is a promising intervention to enhance cognitive, behavioral, and physical performance, though further research is essential to confirm specific effects and address variability. AdipoRon, a synthetic agonist of adiponectin receptors AdipoR1 and AdipoR2, has garnered significant attention for its potential therapeutic effects on cognitive function and behavioral outcomes [40]. Our meta-analysis offers valuable insights into these effects, particularly concerning enhanced cognitive and behavioral performance and improved locomotor activity and task efficiency. Our findings reveal that AdipoRon administration significantly improves various cognitive and behavioral parameters. For instance, in tests assessing novel object preference and time spent in the novel arm or target quadrant, AdipoRon-treated groups exhibited superior performance compared to controls. These findings suggest that AdipoRon positively influences spatial memory and cognitive function. Such enhancements are crucial, especially in conditions characterized by cognitive deficits, such as AD and Type 2 Diabetes Mellitus (T2DM). Supporting these observations, Zhao et al. [117] demonstrated that AdipoRon ameliorates synaptic dysfunction and inhibits tau hyperphosphorylation through the adiponectin receptor/AMPK/mTOR pathway in T2DM mice. The study reported significant restoration of cognitive deficits, including shorter escape latency and increased time spent in the target quadrant, aligning with the meta-analysis’ outcomes. Furthermore, Chu et al. [118] investigated the role of adiponectin deficiency in cognitive dysfunction among obese mice following sevoflurane exposure. The study found that AdipoRon administration partially prevented cognitive impairments, highlighting its neuroprotective properties. The meta-analysis also indicates that AdipoRon enhances locomotor activity and task efficiency. This is evidenced by increased total arm entries and reduced escape latency in behavioral tasks, suggesting heightened motivation and improved physical performance. Azizifar et al. [119] explored the effects of intranasal AdipoRon in a Parkinson’s disease (PD) rat model. The study found that AdipoRon exhibited anxiolytic and antidepressant effects and improved motor function, as evidenced by performance in the open field and elevated plus maze tests. Additionally, Lee et al. [120] reported that chronic AdipoRon treatment mimics the effects of physical exercise by restoring hippocampal neuroplasticity in diabetic mice. The treatment increased progenitor cell proliferation and neuronal differentiation in the hippocampal dentate gyrus, improving cognitive and motor functions. The beneficial effects of AdipoRon on cognitive and behavioral functions are primarily mediated through activating the adiponectin receptor/AMPK/mTOR signaling pathway. Activation of this pathway enhances synaptic plasticity, reduces neuroinflammation, and promotes neurogenesis, all essential for cognitive health [117]. Moreover, AdipoRon has been shown to modulate the expression of synaptic proteins such as Postsynaptic Density Protein 95 (PSD-95), Alpha-Synuclein (SYN), Growth-Associated Protein 43 (GAP43), and Synaptophysin (SYP), which are crucial for synaptic integrity and function. By preserving synaptic structure and function, AdipoRon contributes to improved cognitive and behavioral outcomes [117]. In summary, the meta-analysis, in conjunction with findings from recent studies, underscores the potential of AdipoRon as a therapeutic agent for enhancing cognitive and behavioral functions. Its ability to improve locomotor activity and task efficiency further highlights its multifaceted benefits. These effects are primarily attributed to activating the adiponectin receptor/AMPK/mTOR pathway, enhancing synaptic plasticity and neuroprotection. Future research should focus on elucidating the long-term impacts of AdipoRon and its potential applications in clinical settings for the treatment of cognitive impairments associated with metabolic and neurodegenerative disorders.

It is essential to note that the included studies, besides being similar, contained several differences. Sun et al. [98] and Khandelwal et al. [100] compared the effects of AdipoRon in the same mouse model of AD. However, they assessed different molecular targets, and Khandelwal et al. emphasized the impact of AdipoRon treatment against GLUT4 impairments, which are critical aspects of AD occurrence. Insulin is essential in long-term neuroprotection, and it is known that insulin absence leads to neurodegeneration. IR, which conditions cells to react poorly to insulin signaling, is a key feature of AD occurrence and a significant risk factor for the disease. IR leads to neural and cognitive abnormalities in the brain, predisposing it to cognitive dysfunction through hyperinsulinemia, impaired insulin signaling, and altered Amyloid Precursor Protein (APP) metabolism [121,122,123,124]. Previous research has also indicated that fluctuations in Glucose Transporter Proteins (GLUT) are associated with brain disturbances linked to AD, which may be partially caused by a significant down-regulation of GLUT, most closely associated with IR in the brain. Some authors affirm that the first signal of AD is the lack of GLUT4 translocation [125,126,127]. Ng et al. [101] have also discussed insulin sensitivity in the context of AD and AdipoRon treatment. Their results were like the others, with AdipoRon being a fundamental factor for decreasing IR and augmenting insulin signaling and efficiency. However, these authors did not directly investigate GLUT translocation. Instead, they revealed information about neuroinflammation in the context of insulin signaling restoration. Brain IR causes synaptic dysregulation by impaired axonal transport, apoptosis, Aβ production, and neuroinflammation [128,129]. Particularly, hippocampal IR and inflammation are associated primarily with memory impairments and impaired executive functions [130,131]. This mechanism is proposed to be the primary mediator between peripheral IR and brain dysfunction during AD occurrence [132,133,134,135]. As confirmed by Ng et al., NF-kB levels are critical to AD occurrence. NF-kB facilitates the autocrine production of constitutive AD factors. NF-kB regulates the genetic signal for amyloid plaque production, neurofibrillary tangle occurrence, neuronal apoptosis, and neuroinflammation development [136,137,138,139].

## 5. Conclusions

**Figure 10:**
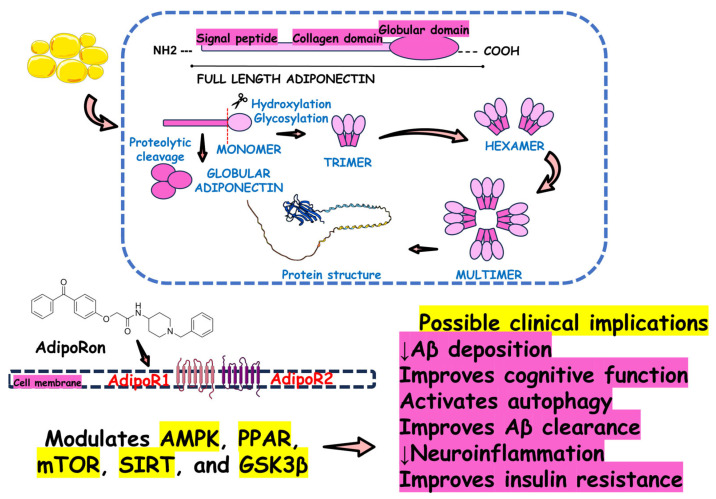
Adiponectin synthesis and effects of AdipoRon against Alzheimer’s Disease. Adiponectin from adipose tissue presents several neuroprotective mechanisms that influence the activity of Adiponectin Receptor 1 (AdipoR1) and Adiponectin Receptor 2 (AdipoR2) receptors in the central nervous system. Since AdipoRon is an adiponectin receptor agonist, this molecule is expected to show the same neuroprotective effects. The evidence suggests that while modulating AdipoR1 and AdipoR2, AdipoRon affects Adenosine Monophosphate-Activated Protein Kinase (AMPK), Peroxisome Proliferator-Activated Receptor (PPAR), Mammalian Target of Rapamycin (mTOR), Sirtuin (SIRT), and Glycogen Synthase Kinase 3 Beta (GSK3β) signaling pathways. By acting on these signaling pathways, AdipoRon can exert many beneficial effects against Alzheimer’s disease, improving Amyloid-Beta (Aβ) deposition and clearance, enhancing cognitive function and brain insulin resistance, and activating autophagy.

The collective body of research highlights that AdipoRon, an adiponectin receptor agonist, exhibits significant neuroprotective effects across various AD models (Figure 10). AdipoRon’s therapeutic potential appears multifaceted, as it engages in several critical pathways contributing to its efficacy. Specifically, AdipoRon has been shown to activate AMPK, a key regulator of cellular energy homeostasis, which can influence various downstream processes essential for maintaining neuronal health. The enhancement of glucose metabolism facilitated by AdipoRon also plays a crucial role in addressing the metabolic dysfunction commonly observed in AD, potentially improving neuronal energy availability and function. Additionally, AdipoRon’s effects on reducing tau phosphorylation—a hallmark of AD pathology—suggest that it may help mitigate one of the critical drivers of neurodegeneration. AdipoRon could help prevent tau aggregation and subsequent neurofibrillary tangle formation, central to cognitive decline in AD, by targeting tau hyperphosphorylation. Furthermore, AdipoRon’s ability to regulate autophagy is significant, as autophagic dysfunction is implicated in the accumulation of toxic protein aggregates and cellular debris, exacerbating neurodegenerative processes.

### Limitations and Future Perspectives

However, there are significant limitations in the current body of research that need to be addressed to validate AdipoRon’s therapeutic potential against AD fully. One such limitation is the reliance on specific animal models, which, while valuable, may not fully represent the complexity and variability of human AD. Differences in genetic backgrounds, disease progression, and comorbidities can affect how well these models translate to human outcomes [140,141,142,143,144,145]. Expanding the duration of treatment in the existing studies also poses a limitation. Many studies have used relatively short treatment periods, which may not capture the long-term effects and safety of AdipoRon. Given the chronic nature of AD, it is crucial to investigate the long-term efficacy and safety of AdipoRon over extended periods to determine its potential for sustained benefit and to monitor for any delayed adverse effects. Additionally, some of the research has been conducted in vitro, which, while providing valuable insights into cellular mechanisms, does not fully replicate the complexity of living organisms. In vivo studies and clinical trials are necessary to confirm the findings from in vitro experiments and to evaluate how AdipoRon performs in a more physiologically relevant context. Addressing these limitations through future research will be crucial for advancing the development of AdipoRon as a viable therapeutic option for AD. This includes expanding studies to encompass a broader range of animal models and patient populations, evaluating long-term treatment outcomes, and conducting robust clinical trials to assess the safety and efficacy of AdipoRon in diverse human populations. Such comprehensive research efforts will help establish AdipoRon’s role in AD treatment and contribute to its potential translation into clinical practice.

First, expanding studies to include a broader range of animal models and genetic backgrounds can help determine the generalizability of AdipoRon’s effects across different AD phenotypes and stages [67,68,146,147,148,149]. This would provide a more comprehensive understanding of how AdipoRon interacts with the varying pathological features of the disease. Second, research should explore optimal dosing regimens and long-term treatment protocols to assess the durability and sustained efficacy of AdipoRon. Investigations into the pharmacokinetics and pharmacodynamics of AdipoRon could offer insights into effective dosing strategies and potential interactions with other medications commonly used in Alzheimer’s treatment. Third, given the promising preclinical results, there is a pressing need for well-designed clinical trials to evaluate the safety and efficacy of AdipoRon in human populations. These trials would focus on assessing cognitive outcomes, biomarkers of neuroinflammation, tau phosphorylation, and autophagy in patients with different stages of AD. Moreover, clinical studies should consider diverse patient populations to understand the drug’s effects across genetic and environmental backgrounds. Additionally, future research could investigate the development of user-friendly delivery systems for AdipoRon, such as oral formulations or transdermal patches, which would improve patient adherence and reduce the treatment burden. Assessing the impact of such delivery methods on drug absorption, efficacy, and side effects is crucial for translating preclinical successes into clinical practice. Lastly, exploring the potential synergistic effects of AdipoRon in combination with other therapeutic agents, such as existing Alzheimer’s drugs or novel compounds, could enhance treatment outcomes. Investigating how AdipoRon interacts with other treatments may uncover new strategies for combination therapies that address multiple aspects of Alzheimer’s pathology.

In addition to the limitations discussed, other potential concerns warrant consideration. One significant aspect is the possibility of AdipoRon interacting with receptors beyond the adiponectin receptor [80,87,88,150,151]. Although the primary mechanism of action described involves AdipoR1/R2 and related pathways, AdipoRon may influence or interact with other cellular receptors or signaling pathways. For instance, interactions with insulin receptors or other metabolic signaling pathways could introduce unforeseen effects, particularly in complex diseases like Alzheimer’s, where metabolic dysregulation is prevalent. Such interactions could enhance therapeutic efficacy or contribute to unintended side effects, which may complicate clinical application. Moreover, the potential for off-target side effects or interactions with other medications used in the treatment of Alzheimer’s or related conditions must be carefully evaluated. As AdipoRon may affect multiple cellular processes, its use in combination with other drugs could lead to interactions that alter the efficacy or safety profile of the treatment. For example, concurrent use with anti-inflammatory or neuroprotective agents might result in synergistic effects or, conversely, create adverse interactions. Thus, a thorough investigation into potential drug-drug interactions is essential to ensure that AdipoRon can be safely and effectively integrated into existing therapeutic regimens.

In conclusion, the collective evidence from these studies underscores the potential of AdipoRon as a promising therapeutic agent for AD, highlighting its multifaceted benefits across various molecular pathways. The findings suggest that AdipoRon may offer significant neuroprotection by enhancing Aβ clearance, improving cognitive function, reducing tau hyperphosphorylation, and mitigating neuroinflammation. Despite the promising results, each study presents limitations, including the reliance on specific animal models and the need for validation in diverse and long-term human trials. Addressing these limitations through further research will be crucial in determining the clinical viability of AdipoRon and its potential to modify AD progression while addressing both symptomatic relief and underlying pathophysiological mechanisms.

## Figures and Tables

**Table 1:** AdipoRon-based adiponectin replacement therapy against Alzheimer’s disease.

Refs.	Type of Study	Model (s)	Experimental Group (s) and Treatment’s Option and Duration	Intervention (s) (Concentration or Dose)	Outcome (s)	Pathway (s)
Sun et al., 2024 [98]	In vitro and in vivo	HT22 cells, APP/PS1 mice	In vitro: AdipoRon 48 h; in vivo: AdipoRon, 7 days	1 mM in vitro, 1 μg in vivo	↓Aβ deposition, ↑cognitive function, ↑autophagy, ↑Aβ clearance	↑AdipoR1/AMPK-dependent nuclear translocation of GAPDH, ↑SIRT1 activation, ↓DBC1 interaction with SIRT1
Wang et al., 2023 [99]	In vitro and in vivo	SH-SY5Y cells, P301S mice	In vitro: AdipoRon 48 h; in vivo: AdipoRon/corn oil, 4 months	20 μM in vitro, 50 mg/kg in vivo	↓Hyperphosphorylated tau, ↓memory deficits, ↓synaptic deficiencies, ↑mitochondrial fusion	↑AMPK/GSK3β activation, ↓tau (S396, T231) in vitro, ↑Mfn2, OPA1, ↑AMPK/SIRT3, ↑GSK3β in vivo
Khandelwal et al., 2022 [100]	In vitro and in vivo	Neuro2A cells, APP/PS1 mice	In vitro: AdipoRon 12 h; in vivo: AdipoRon/DMSO, 30 days	10 μM in vitro, 50 mg/kg in vivo	↑Glucose uptake, ↓IR, ↑insulin sensitivity, ↓cognitive deficits, ↓Aβ deposition, ↓neuroinflammation	↑GLUT4 translocation, ↑AKT, GSK3β, AMPK phosphorylation in vitro, ↑GLUT1, GLUT4, ACC, PGC1α, APOE, PSD-95, SYP in vivo
Ng et al., 2021 [101]	In vitro and in vivo	HT-22 cells, 5xFAD mice	In vitro: AdipoRon 48 h; in vivo: AdipoRon/corn oil, 3 months	5 μM in vitro, 50 mg/kg in vivo	↑Neuronal insulin-signaling, ↑insulin sensitivity, ↑spatial/neuronal/synaptic memory, ↑learning, ↓neuroinflammation	↑AMPK, ↓BACE1, NF-κB in vitro, ↑AKT, GSK3β phosphorylation, ↓IRS-1, Aβ, BACE1, APPβ, βCTF, IL-1β, TNF-α in vivo
He et al., 2021 [102]	In vitro and in vivo	N2a/APPswe cells, 5xFAD, APP/PS1, APN KO mice	In vitro: AdipoRon, 3-MA, CQ 24 h; in vivo: AdipoRon 2–4 months	1 or 5 μM in vitro, 50 mg/kg in vivo	↓Aβ deposition, ↓neuroinflammation, ↓cognitive impairment, ↓spatial memory deficits, ↑autophagy, ↑lysosomal activity	↑AMPK-mTOR activation, ↑AdipoR1, APPL1 expression
Liu et al., 2020 [93]	In vitro and in vivo	NE-4C cells, APP/PS1 mice	In vitro: AdipoRon 48 h; in vivo: AdipoRon/vehicle injection, 7 days	1 μM in vitro, 1 μg in vivo	↓Aβ-induced neuronal injury, ↑cell viability, ↑cognitive function, ↓Aβ deposition	↑NSC proliferation, ↑AMPK, CREB activation, ↓ΔΨm dissipation in vitro and in vivo

**Abbreviations:** ↑, Increase; ↓, Decrease; 3-MA and CQ, Autophagy Inhibitors; ΔΨm, The Changes in Relative Mitochondrial Membrane Potential; βCTF, β-C-Terminal Fragment; ACC, Acetyl-CoA Carboxylase; AdipoR1, Adiponectin Receptor 1; Aβ, Amyloid-Beta; AMPK, Adenosine Monophosphate-Activated Protein Kinase; AKT, Protein Kinase B; APOE, Apolipoprotein E; APPβ, Amyloid Precursor Protein Beta; APPL1, Adaptor Protein, Phosphotyrosine Interacting with PH Domain and Leucine Zipper 1; BACE1, Beta-Secretase 1; CREB, Cyclic Adenosine Monophosphate (cAMP)-Response Element Binding Protein; DBC1, Deleted in Breast Cancer 1; DMSO, Dimethyl Sulfoxide; GAPDH, Glyceraldehyde-3-Phosphate Dehydrogenase; GLUT1, Glucose Transporter 1; GLUT4, Glucose Transporter 4; GSK3β, Glycogen Synthase Kinase 3 Beta; IL-1β, Interleukin-1 Beta; IR, Insulin Resistance; IRS-1, Insulin Receptor Substrate 1; Mfn2, Mitofusin 2; mTOR, Mammalian Target Of Rapamycin; NF-κB, Nuclear Factor Kappa B; NSC, Neural Stem Cells; OPA1, Optic Atrophy 1; PGC1α, Peroxisome Proliferator-Activated Receptor-Gamma Coactivator 1; PSD-95, Postsynaptic Density 95; SIRT1, Sirtuin 1; SIRT3, Sirtuin 3; SYP, Synaptophysin; TNF-α, Tumor Necrosis Factor Alpha.

## Data Availability

No new data were created or analyzed in this study. Data sharing does not apply to this article.
